# A 14-Day Sleep Hygiene Intervention Improves Aerobic Performance and Reduces Anticipatory Cortisol in University Soccer Players

**DOI:** 10.3390/sports14050179

**Published:** 2026-04-29

**Authors:** Adele Broodryk, Retief Broodryk

**Affiliations:** Physical Activity, Sport and Recreation Research Focus Area (PhASRec), Faculty of Health Sciences, North-West University, Potchefstroom 2520, South Africa; retief.broodryk@nwu.ac.za

**Keywords:** sleep hygiene, recovery, soccer, dual-career athlete, females, males, repeated sprint ability, aerobic capacity, cortisol

## Abstract

**Background:** Sleep is a critical recovery mechanism for athletes, supporting hormonal regulation, muscle repair, and cognitive function. Dual-career athletes are particularly vulnerable to sleep disruption, which may impair performance and stress regulation. This study examined the effects of a 14-day sleep hygiene intervention protocol (SHIP) on aerobic and anaerobic performance, as well as anticipatory cortisol responses, in university-level soccer players. **Methods:** Thirty athletes (females: *n* = 14, 22.1 ± 3.3 y, 157.8 ± 6.0 cm, 53.5 ± 3.9 kg, males: *n* = 16, 21.5 ± 1.7 y, 167.5 ± 5.9 cm, 62.7 ± 5.4 kg) completed the Pittsburgh Sleep Quality Index (PSQI), provided pre-test salivary cortisol samples, and performed the Yo-Yo Intermittent Recovery Test Level 1 (YYIR1) and Repeated Anaerobic Sprint Test (RAST) before and after the intervention (adhering daily to 10–18 individualized sleep hygiene). **Results:** The SHIP significantly reduced sleep latency (*p* = 0.04) and increased sleep duration (*p* = 0.03), and PSQI scores (*p* < 0.001) in both sexes. Females showed marked increases in sleep duration (*p* = 0.002), while males showed improved latency (*p* = 0.07). Five behaviourally coherent clusters derived from the SHIP adherence explained a substantial proportion of variance (74.99%). Stimulant and metabolic regulation, and bedroom light and thermal environment control consistently predicted sprint and physiological outcomes (*p* < 0.05). Anticipatory cortisol decreased before both tests (*p* = 0.03–0.04). YYIR1 performance improved for the full cohort (*p* = 0.001). RAST times slowed slightly (*p* = 0.02), though fatigue index improved (*p* = 0.05). **Conclusions:** A short-term SHIP effectively enhanced sleep, reduced physiological stress, and improved key performance outcomes in collegiate athletes.

## 1. Introduction

Sleep is increasingly recognized as a vital component of health, performance, and recovery in athletes across various sports [1,2,3,4,5,6]. Sufficient sleep duration and quality are essential for restoring physical abilities, consolidating learning, regulating mood, and maintaining hormonal balance [1,3,4,7,8]. In high-intensity, intermittent sports like soccer, where both aerobic and anaerobic energy systems are repeatedly challenged, poor-quality or inadequate sleep can impair recovery, raise fatigue levels, and affect performance [5]. Therefore, healthy sleep habits can be implemented to improve sleep quality and counter the negative effects associated with inadequate sleep, potentially supporting health and performance [9].

The National Sleep Foundation recommends 7–9 h of sleep for adults and 8–10 h for adolescents [6]. However, these guidelines are based on general population health and may underestimate the needs of athletic populations. Indeed, athletes often require greater sleep duration to support recovery, adaptation, and performance, with evidence suggesting that elite athletes benefit from ≥9 h of sleep per night [1,10,11]. This was confirmed by a study done on elite athletes, reporting a mere 6.7 h of sleep, though needing 8.3 h to feel fully rested [12]. While much of the early literature has focused predominantly on sleep duration, there is increasing recognition that sleep quality is an equally important, and often overlooked, determinant of recovery and performance outcomes [2,13]. Sleep quality encompasses multiple dimensions, including sleep latency, sleep disturbances, efficiency, and subjective sleep perception, which may not be adequately captured by duration alone [14]. Indeed, athletes may achieve recommended sleep durations while still experiencing poor sleep quality, which can impair recovery processes and subsequent performance [15].

Soccer is a demanding sport and requires the simultaneous use of both aerobic and anaerobic energy systems, whilst performing multiple actions involving change in directions [16,17]. Over a 90 min match, players typically cover 10–12 km, with 10–15% thereof performed repetitively at high intensity [18]. Aerobic capacity is essential for sustaining prolonged activity, supporting recovery between high-intensity bouts, and maintaining tactical positioning [17,19]. Anaerobic capacity underpins explosive actions such as sprinting, jumping, and rapid changes in direction, movements central to offensive and defensive play [16,17,18]. Fatigue in either energy system can compromise technical execution, tactical decision-making, and overall performance efficiency [17]. Consequently, interventions that enhance recovery and reduce fatigue across both energy systems are of particular interest in soccer performance research.

University soccer players are expected to comply with both academic and sports demands. This typically includes early training, evening competitions, travel, social obligations, late-night screen use, and intensive coursework [4,5,10,11,20,21]. These factors often disrupt sleep routines, resulting in irregular sleep patterns, and reduced sleep duration and quality [5,21,22]. The consequences of insufficient sleep in athletic populations are multifaceted, potentially affecting performance, injury risk, and academic success [1,3,9,20]. Physiologically, inadequate sleep disrupts anabolic-catabolic hormonal balance, impairs glycogen resynthesis, and delays muscle repair, thereby prolonging recovery from both training and competition [5]. Psychologically, sleep restriction increases perceived exertion, impairs mood stability, and compromises cognitive functions such as decision-making, attention, and working memory [1,23]. These factors are particularly critical in soccer, a sport that requires sustained aerobic output alongside rapid anaerobic bursts, as well as constant tactical awareness and decision-making under pressure [5,16,17,18]. Poor sleep, therefore, poses a direct threat to both the physical and cognitive demands inherent to soccer performance [5].

A study by Camargo and colleagues [24] found that in female collegiate soccer players, both sleep duration and subjective sleep quality significantly predicted psychological well-being and perceived exertion throughout a competitive season. Similarly, Moen et al. [22] reported that match play, particularly late-evening games at a female national level, was associated with a marked reduction in total sleep time and disruptions in sleep architecture, including decreased rapid eye movement (REM) and slow-wave sleep, stages critical for motor learning and physical recovery. Comparative studies further indicate that female collegiate athletes report poorer sleep quality and shorter sleep duration compared to male peers, suggesting that gender-specific and contextual factors may exacerbate the sleep challenges faced by women in university sport [4]. These findings are supported by evidence from Mong and Cusmano [25], who demonstrated that biological gender differences, including hormonal fluctuations across the menstrual cycle, can influence sleep regulation, circadian rhythms, and sleep architecture, potentially increasing vulnerability to sleep disturbances in females.

The relationship between sleep and hormonal regulation provides another layer of importance. Cortisol, a glucocorticoid hormone secreted by the adrenal glands [26], is central to the body’s stress response and recovery processes [27]. It follows a diurnal rhythm, typically peaking shortly after waking and gradually declining throughout the day [26,27]. In athletes, elevated cortisol levels, particularly in anticipation of competition or training, can impair recovery, increase fatigue, and blunt performance [28]. Poor sleep quality and insufficient duration have both been linked to dysregulation of cortisol secretion [26,27]. For instance, research has demonstrated that even modest sleep restriction can elevate cortisol concentrations, especially in the morning and pre-exercise periods [23,27]. Such disruptions may increase catabolic activity, hinder muscle repair, and aggravate perceived exertion [28], emphasizing the critical role of sleep in hormonal regulation.

One avenue to mitigate these challenges is the implementation of sleep hygiene practices [29]. Sleep hygiene refers to a set of behavioural and environmental strategies designed to promote consistent, restorative sleep [29,30]. Common recommendations include maintaining a regular bed- and wake time, reducing pre-sleep screen exposure, avoiding caffeine and heavy meals in the evening, and ensuring a quiet, dark, and cool sleep environment, to name a few [10,30,31]. For athletes, additional strategies such as post-training relaxation routines, light exposure management, and mindfulness-based approaches may enhance the effectiveness of these practices [31,32]. Despite their simplicity and relatively low cost, many athletes remain unaware of or fail to implement sleep hygiene strategies consistently [10].

Recent studies demonstrated that structured and media-based sleep hygiene programmes enhance sleep efficiency and recovery, translating into improved physical outputs during competition and training [19,32]. Complementing these findings, systematic reviews and applied studies emphasize that personalized, behaviour-focused strategies not only optimize sleep duration and quality but also assist endurance, neuromuscular function, and perceived well-being across diverse athletic contexts [10,30,33,34]. However, this body of evidence remains limited, particularly in dual-career male and female athletes, where contextual challenges such as menstrual cycle fluctuations, hormonal influences, and academic stress may alter the effectiveness of interventions [35].

Despite the growing recognition of sleep as a critical factor in athletic recovery and performance, significant gaps remain in the literature. Existing research focuses primarily on descriptive sleep patterns or the effects of acute sleep deprivation, while only a minority of studies systematically evaluate the effectiveness of structured sleep hygiene intervention protocols (SHIP) in applied sporting contexts [32,34]. Furthermore, limited research has simultaneously examined both physiological outcomes, such as aerobic and anaerobic fatigue, and hormonal responses, particularly cortisol regulation, following such interventions in both male and female athletes [33,36]. This gap is especially evident in university-level athletes, a population at high risk for sleep disruption due to the combined demands of academic and athletic commitments, yet one that remains underrepresented in intervention-based research [7,19,21,35]. In addition, although emerging evidence suggests that female athletes may experience greater sleep disturbances due to biological and contextual factors, including hormonal fluctuations, sex-specific responses to sleep hygiene interventions remain poorly understood [4,25,35].

Therefore, there is a need for research that evaluates practical, low-cost sleep hygiene strategies and their combined effects on performance-related fatigue and hormonal responses in both male and female university soccer players. This study addresses this gap by investigating the impact of a structured sleep hygiene protocol on aerobic and anaerobic fatigue, as well as anticipatory cortisol reactivity, in this population.

## 2. Materials and Methods

### 2.1. Study Design

A randomized counterbalanced repeated-measure crossover trial was implemented over three weeks. Data collection encompassed questionnaires, physical performance assessments, and systematic observations throughout the intervention period. Ethical approval and informed consent were obtained (NWU-00299-21-A1) before data collection per the Declaration of Helsinki.

### 2.2. Participants

A total of 30 collegiate soccer players (females: *n* = 14, 22.1 ± 3.3 y, 157.8 ± 6.0 cm, 53.5 ± 3.9 kg, males: *n* = 16, 21.5 ± 1.7 y, 167.5 ± 5.9 cm, 62.7 ± 5.4 kg) from a tertiary institution took part in the study and provided written consent before testing. To achieve adequate statistical power, an a priori sample size of 35 participants was required based on the following inputs: (i) repeated measures within-between interaction, (ii) anticipated effect size of f = 0.25, (iii) type-1 error rate of 5%, (iv) type-2 error rate of 20%, (v) a total of 2 repeated measurements per condition (i.e., SHIP vs. no-SHIP), and (vi) an expected correlation of 0.70 among repeated measures [37]. However, data from five participants were excluded from further analysis due to incomplete physical or cortisol testing.

### 2.3. Instrumentation and Measurements

#### 2.3.1. Pittsburgh Sleep Quality Index (PSQI)

Sleep quality was assessed using the Pittsburgh Sleep Quality Index (PSQI), a validated questionnaire comprising 19 items that generate seven component scores: subjective sleep quality, sleep latency, sleep duration, habitual sleep efficiency, sleep disturbances, use of sleep medication, and daytime dysfunction [13]. Each component is scored on a 0–3 scale, and component scores are summed to yield a global PSQI score ranging from 0 to 21, with higher scores indicating poorer sleep quality. A global PSQI score > 5 is indicative of clinically relevant poor sleep quality [13]. The questionnaire was adapted by participants recalling their sleep over the last seven days, and not 30 days as per the original PSQI.

#### 2.3.2. Physical Performance Tests

##### Yo-Yo Intermittent Recovery Test Level 1 (YYIR1)

This test measures aerobic performance, requiring participants to repeatedly sprint between short intermittent recovery periods [38], making it particularly relevant for soccer, where aerobic capacity underpins repeated sprint ability interspersed with brief recovery phases. The test was conducted on a standard grass soccer field with players wearing boots. The test involved 20 m shuttle runs paced by audio signals, with 10 s active recovery between each shuttle. The recorded parameters comprised total distance covered, number of shuttles completed and maximum heart rate (HRmax) and rating of Perceived exertion (RPE). HRmax was measured at test termination using a Polar H10 heart rate monitor and chest strap. RPE was obtained 5 min post-test termination on a 6–20 Likert scale [39].

##### Repeated Anaerobic Sprint Test (RAST)

Anaerobic capacity and fatigue were assessed using the RAST protocol [40]. The test comprises six 20 m out and back sprints (totalling 40 m), interspersed with 20 s passive recovery. A baseline 40 m sprint was recorded using a photocell system (SmartSpeed, Fusion Sport, Brisbane, QLD, Australia), followed by a 5 min recovery. The test was to be repeated if the first sprint exceeded baseline by >2.5%, although this did not occur. Performance metrics included fastest- and mean repetition times, and percentage decrements over the six repetitions (fatigue index), while HRmax and RPE measurements mirrored the procedures of the YYIR1 test.

#### 2.3.3. Saliva Sampling

Saliva samples were collected for the assessment of cortisol by using the passive drool test [41]. The mouth was rinsed thoroughly with lukewarm water ten minutes before sampling to remove any food substances. If needed, the subjects chew on a piece of Parafilm (Bemis Company, Inc., Oshkosh, WI, USA) to stimulate saliva flow, which was collected through a plastic straw into a 2 mL vial. Samples were stored in a freezer (−80 ± 1 °C) until analyzed by a professional laboratory. The cortisol content from 20 µL saliva samples was determined by using the Salimetrics^®^ High Sensitivity Salivary Cortisol Enzyme Immunoassay Kit (Salimetrics, LLC., State College, PA, USA). The samples were transferred into a Berthold luminometer (Bemis Company, Inc., Mannheim, Germany) to calculate the average relative luminescence units, which were plotted against the concentration to determine the exact value. There are both linear and non-linear correlation coefficients (r = 0.8–1.0) between saliva and serum cortisol values based on this method. This specific biochemical analysis has an intra-assay variation ranging between 0.4 and 1.7% and inter-assay variation between 0.8 and 1.8% [42]. To minimize the effect of external factors, players were advised to be awake and fasted for an hour before sampling. Owing to the diurnal rhythm of cortisol in the body, samples were collected at the same time (at precisely 14:00) for all testing periods [42], 30 min before each test to measure the stress response [43].

#### 2.3.4. Sleep Hygiene Intervention Protocol (SHIP)

During the no-SHIP conditions, players followed their normal sleep routine in their home setting, and thus, were allowed to self-regulate electronic use, bedtime, pre-bed light (i.e., 60 ± 12 Watts), and regular sleeping patterns. The experimental condition (i.e., SHIP) was completed over 14 days, also in their home setting. Participants were required to adhere to at least 10 of the following sleep hygiene recommendations as set out in “Table 1” based on their preference and environment [44], whilst documenting their daily adherence in a sleep diary. The sleep hygiene components were selected based on established evidence and a prior systematic evaluation [45]. Readers are referred to this work for a detailed rationale supporting each component.

### 2.4. Procedures

**Figure 1:**
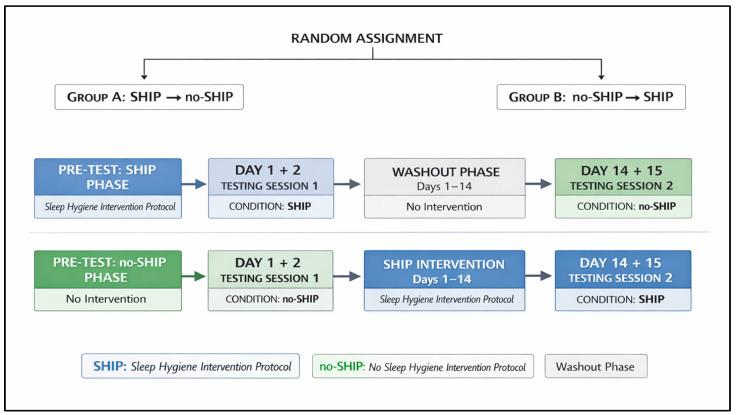
Randomized counterbalanced crossover trial. Participants were randomly allocated to one of two sequences: SHIP followed by no-SHIP, or no-SHIP followed by SHIP. Each phase lasted 14 days and was separated by a 14-day washout period to minimize potential carryover effects. Testing sessions were conducted at the end of each phase under standardized conditions.

A randomized counterbalanced repeated-measure crossover trial was employed, in which participants were randomly allocated to one of two sequences: (1) sleep hygiene intervention protocol (SHIP) followed by no intervention (no-SHIP), or (2) no-SHIP followed by SHIP (Figure 1).

In Sequence 1, participants completed a 14-day SHIP before the first testing session, followed by a 14-day washout period (no intervention), and then underwent testing under the no-SHIP condition. In Sequence 2, participants initially completed testing under the no-SHIP condition, followed by a 14-day SHIP and a second testing session. The washout period was included to minimize potential carryover effects. All testing procedures were conducted under standardized conditions and repeated at the same time of day across experimental phases, aligned with participants’ habitual training times, to minimize the influence of circadian rhythms, individual chronotype, and time-of-day training preferences on performance outcomes. On Day 1 of each testing phase, participants completed the PSQI, followed by anthropometric assessments (body mass and stature) and resting heart rate measurement. A saliva sample was collected via passive drool before a standardized 15 min dynamic warm-up. Participants then performed the RAST on Day 1 and the YYIR1 on Day 2. HRmax and RPE were recorded following each test. Participants were instructed to abstain from caffeine, alcohol, and strenuous exercise for 48 h before testing and fast for at least one hour before saliva collection. Throughout the study, participants were required to maintain consistent dietary, training, and lifestyle habits and to record daily adherence to each sleep hygiene component during the intervention phase. To minimize the influence of academic-related stress, all testing was conducted at the start of the academic term during the pre-season periodization phase.

### 2.5. Statistical Analyses

A principal component analysis (PCA) was conducted to identify clustering patterns among the 18 sleep hygiene recommendations and to reduce the data to conceptually meaningful components. Before extraction, sampling adequacy was evaluated using the Kaiser–Meyer–Olkin (KMO) measure and Bartlett’s Test of Sphericity. Components were retained based on eigenvalues greater than 1.0 and inspection of the scree plot. Factor extraction was performed using the principal component method, followed by varimax rotation with Kaiser normalization to improve interpretability. Rotated factor loadings ≥ 0.40 were considered meaningful and used to determine component membership. Communalities were examined to ensure that retained components adequately explained variance within each variable. Component scores were saved using the regression method and used as predictors in subsequent analyses. Internal consistency of each component was evaluated using Cronbach’s alpha, where applicable.

Primary analyses used linear mixed-effects models with fixed effects for Condition, Gender, their interaction, and (for females) menstrual phase, plus a random intercept for participant. Where assumptions were met, we additionally report two-way repeated-measures ANOVA results (Condition × Gender) for comparability with conventional reporting. Model estimates were reported as fixed-effect coefficients with 95% confidence intervals. For outcomes involving more than one pairwise comparison (e.g., sprint splits, cortisol timepoints), multiple-comparison correction was applied using the Holm–Bonferroni procedure within each outcome family (anaerobic, aerobic, and endocrine variables) to reduce the risk of type I error. Effect sizes were expressed as Hedges’ g (ESg), interpreted as: trivial (<0.20), small (0.20–0.49), medium (0.50–0.80), large (0.81–1.20), very large (1.21–2.00), and huge (>2.00) [46]. All statistical analyses were performed using IBM SPSS Statistics (version 31, IBM Corp., Armonk, NY, USA) and Jeffreys’s Amazing Statistics Program (JASP, version 0.95.4, JASP Team, Amsterdam, The Netherlands). The significance level was set at *p* < 0.05 (two-tailed).

## 3. Results

### 3.1. Principal Component Analysis of Sleep Hygiene Recommendations

Sampling adequacy for the PCA was acceptable (KMO = 0.537), and Bartlett’s Test of Sphericity indicated that the correlation matrix was suitable for factor analysis (χ^2^ (153) = 337.02, *p* < 0.001). Based on eigenvalues greater than 1.0 and scree plot inspection, five components were retained.

The five clusters extracted explained 74.99% of the total variance. Before rotation, the first cluster accounted for 31.10% of the variance, followed by the second (18.65%), third (9.41%), fourth (8.08%), and fifth (7.75%). Following varimax rotation, variance was more evenly distributed across clusters (21.63%, 14.76%, 13.78%, 13.68%, and 11.14%, respectively). Communalities following extraction ranged from 0.563 to 0.904, indicating that a substantial proportion of variance was explained across all sleep hygiene behaviours. The rotated component matrix (refer to Supplementary Materials) displays factor loadings ≥ 0.40 and was used to define the final cluster structure.

Cluster 1 (Pre-sleep light exposure and device management) comprised behaviours related to reducing visual and electronic stimulation before sleep, including electronic device restriction and blue-light management (SHIP components: 2, 3, 4, 11, 13). Cluster 2 (Sensory and Environmental Sleep Buffering) reflected strategies aimed at minimizing sensory input and environmental disturbances during sleep (SHIP components: 1, 10, 14, 15). Cluster 3 (Stimulant and metabolic regulation) grouped behaviours associated with limiting physiological arousal before bedtime through dietary and metabolic control (SHIP components: 8, 9, 12). Cluster 4 (Bedroom light and thermal environment control) represented optimization of the physical sleep setting through management of lighting and room temperature (SHIP components: 5, 6, 7), while Cluster 5 (Sleep continuity and behavioural regularity) encompassed behaviours supporting consistent sleep timing, reduced nocturnal interruptions, and regular routines (SHIP components: 16, 17, 18).

### 3.2. Sleep Measures

**Figure 2:**
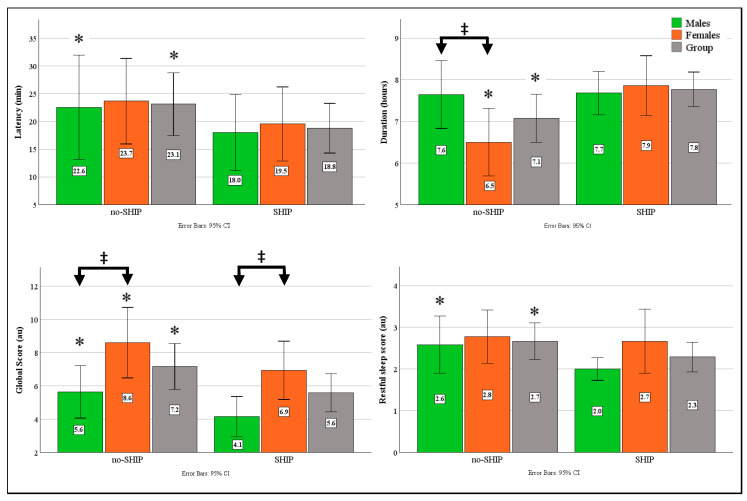
Pittsburgh Sleep Quality results between conditions. CI = Confidence Intervals; SHIP = Sleep Hygiene Intervention Period, au = arbitrary unit, min = minutes, h = hours, ‡ *p* < 0.05 between genders, * *p* < 0.05 between conditions.

The results for sleep measures derived from the PSQI are presented in Figure 2. Before the SHIP, eight participants were considered good sleepers (PSQI < 5), 14 as mild (PSQI 5–10), and 8 as moderate sleepers (PSQI 11–15). Following the SHIP, 13 participants obtained scores below 5 (good sleepers), 16 obtained mild scores, with only one maintaining a moderate score (PSQI > 10).

Following the 14-day SHIP, the entire group displayed significant improvements for sleep latency (*p* = 0.04, ESg = 0.41, CI: 0.02–0.79), sleep durations (*p* = 0.03, ESg = −0.45, CI: −0.83–−0.05), and PSQI scores (*p* < 0.001, ESg = 0.95, CI: 0.87–0.98). The global PSQI scores were significantly different between genders before (*p* = 0.012, ESg = −094, CI = −1.68–−0.19) and after the SHIP (*p* = 0.01, ESg = −1.0, CI: −1.77–−0.22). Both males and females displayed lower PSQI scores following the SHIP (*p* < 0.01, ESg > 0.95). Before the intervention, females slept less compared to the men (*p* = 0.01, ESg = 0.96, CI: 0.21–1.7), which significantly improved post-SHIP (*p* = 0.002, ESg = −1.0, CI: −1.6–−0.34). In contrast, the males improved their sleep latency after the SHIP (*p* = 0.07, ESg = 0.52, CI: −0.05–1.07).

### 3.3. Physical Test Results

#### 3.3.1. RAST Results

**Figure 3:**
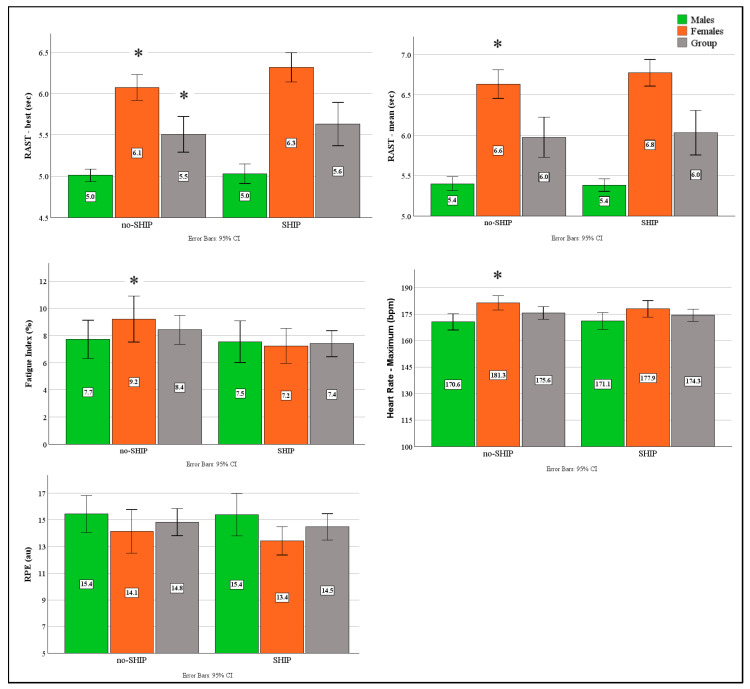
Effects of 14-day SHIP on anaerobic performance metrics across No-SHIP and SHIP Conditions. CI = Confidence Intervals, SHIP = Sleep Hygiene Intervention Period, bpm = beats per minute, au = arbitrary unit, sec = seconds, RAST = Repeated Anaerobic Sprint Test, * *p* < 0.05 between conditions.

As observed from Figure 3, the best RAST times were slower following the SHIP (*p* = 0.02, ESg = −0.46, CI: −0.83–−0.07), which was strongly driven by the performance decrease in the females for both best and average times (*p* < 0.05, ESg > 0.69). The females also displayed significantly lower fatigue index scores (*p* = 0.05, ESg = 0.57, CI: 0.01–1.13) and maximum heart rate values following the SHIP (*p* = 0.05, ESg = 0.59, CI: 0.01–1.15). Linear regression models incorporating cluster classification and experimental factors significantly predicted RAST best sprint time (F (5, 25) = 5.0, *p* = 0.003, adj. R^2^ = 0.50), mean times (F (5, 25) = 4.62, *p* = 0.004, adj. R^2^ = 0.38), and HRmax (F (5, 25) = 6.36, *p* < 0.001, adj. R^2^ = 0.47), indicating that the included predictors explained a moderate-to-large proportion of variance in performance outcomes. At the individual predictor level, RAST best times were influenced predominantly by cluster 3 (*p* = 0.005, β = 0.43), cluster 4 (*p* = 0.027, β = 0.33) and cluster 5 (*p* = 0.005, β = −0.5). RAST mean times were also influenced by cluster 3 (*p* = 0.012, β = 0.39), cluster 4 (*p* = 0.013, β = 0.39) and cluster 5 (*p* = 0.009, β = −0.5). Heart rate responses were significantly influenced by cluster 3 (*p* = 0.006, β = 0.40), cluster 4 (*p* = 0.005, β = 0.41) and cluster 5 (*p* = 0.005, β = −0.41).

Gender-specific regression analyses revealed distinct patterns for the females only. At the group level, all models were significant for best RAST times (F (5, 9) = 5.9, *p* = 0.01, adj. R^2^ = 0.64), mean times (F (5, 9) = 6.1, *p* = 0.01, adj. R^2^ = 0.77), heart rate responses (F (5, 9) = 6.8, *p* = 0.007, adj. R^2^ = 0.79) and RPE (F (5, 9) = 3.57, *p* = 0.047, adj. R^2^ = 0.48). At the cluster level, heart rate was influenced by clusters 3 (*p* = 0.035, β = 26.74) and 4 (*p* = 0.032, β = 24.5), while RPE was only predicted by cluster 4 (*p* = 0.042, β = 3.79).

#### 3.3.2. YYIR-1 Results

**Figure 4:**
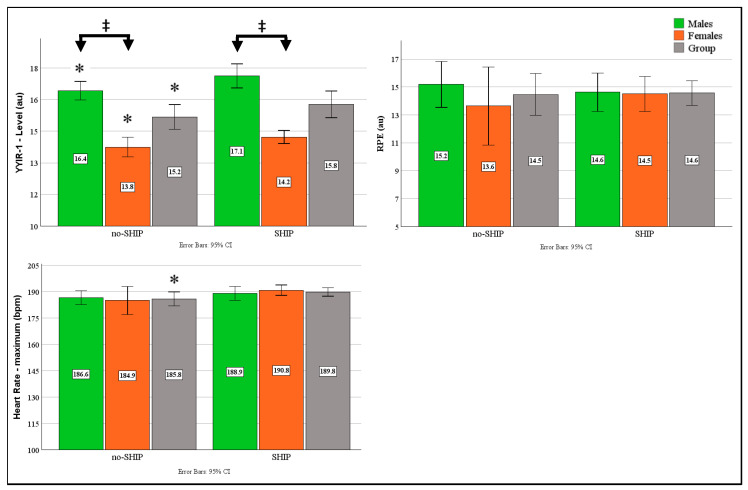
Effects of 14-day SHIP on aerobic performance metrics across No-SHIP and SHIP Conditions. CI = Confidence Intervals, SHIP = Sleep Hygiene Intervention Period, bpm = beats per minute, au = arbitrary unit, ‡ *p* < 0.05 between genders, * *p* < 0.05 between conditions.

The total group executed significantly more YYIR-1 shuttles after the SHIP (*p* = 0.001, ESg = −0.8, CI: −1.2–−0.39), which was driven by increases observed for both males (*p* = 0.005, ESg = −0.81, CI: −1.37–0.24) and females (*p* = 0.009, ESg = −0.82, CI: −1.4–0.19) (Figure 4). Maximum heart rate values also demonstrated significantly higher values for the whole group following the SHIP (*p* = 0.04, ESg = −0.4, CI: −0.76–−0.02). For the females, Cluster 5 was the only significant predictor for YYIR1 performance (*p* = 0.029, β = −0.39), with cluster 1 demonstrating borderline significance (*p* = 0.052, β = −0.34). Additionally, cluster 4 demonstrated significance for RPE scores (*p* = 0.021, β = −0.73) with cluster 3 showing borderline significance (*p* = 0.05, β = −0.6).

### 3.4. Cortisol Results

**Figure 5:**
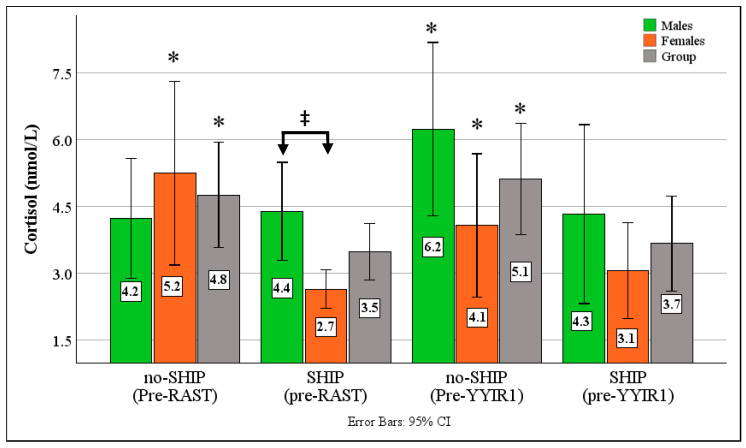
Effects of 14-Day SHIP on Anticipatory Cortisol Responses pre-RAST/YYIR1. SHIP = Sleep Hygiene Intervention Period, RAST = Repeated Anaerobic Sprint Test; YYIR1 = Yo-Yo Intermittent Recovery Test 1; ‡ *p* < 0.05 between genders * = *p* < 0.05 between conditions.

Anticipatory cortisol secretion (Figure 5) for the entire group was noticeably lower following the SHIP before both fatiguing tests (RAST: no-SHIP: 4.79 ± 2.9 vs. SHIP: 3.5 ± 1.57, *p* = 0.03, ESg = 0.43, CI: 0.04–0.8; YYIR-1: no-SHIP: 5.05 ± 3.09 vs. SHIP: 3.7 ± 2.6, *p* = 0.04, ESg = 0.42, CI: 0.03–0.80). Both the male (RAST: no-SHIP: 4.38 ± 2.3 nmol/L vs. SHIP: 4.29 ± 1.7 nmol/L, *p* = 0.8, YYIR-1: no-SHIP: 5.95 ± 3.16 nmol/L vs. SHIP: 4.29 ± 3.09 nmol/L, *p* = 0.4, ESg = 0.50, CI: 0.05–0.81) and female (RAST: no-SHIP: 5.25 ± 3.57 nmol/L vs. SHIP: 2.65 ± 0.75 nmol/L, *p* = 0.01, ESg = 0.79, CI: 0.18–1.38, YYIR-1: no-SHIP: 4.08 ± 2.79 nmol/L vs. SHIP: 3.06 ± 1.87 nmol/L, *p* = 0.08, ESg = 0.5, CI: 0.07–1.04) group displayed lower responses following the sleep intervention period. The females demonstrated significantly lower responses compared to the males before the RAST (*p* = 0.006, ESg = −0.61, CI: −0.81–0.27).

## 4. Discussion

This study investigated the effects of a 14-day structured SHIP on sleep quality, aerobic and anaerobic performance, and anticipatory cortisol responses in male and female university-level soccer players. The SHIP improved sleep outcomes in both genders, with additional gender-specific effects observed for sleep efficiency, performance variables, and cortisol responses. The intervention significantly increased sleep duration, reduced sleep onset latency, and improved global PSQI scores, indicating enhanced sleep efficiency across both groups. These findings support existing evidence that sleep hygiene interventions can meaningfully improve sleep quality, performance, and recovery in athletic populations [2,7,10,12,30,32,33,36,47]. Importantly, these gains in sleep quality were accompanied by measurable changes in physical performance and physiological stress markers, suggesting that improvements in sleep quality were associated with enhanced readiness and reduced anticipatory strain prior to exercise.

Sleep functions not only as a recovery mechanism but also as a modifiable determinant that athletes can leverage to enhance performance. In this regard, improvements in sleep were associated with enhancements in both anaerobic and aerobic performance. Anaerobic outcomes revealed small reductions in best and mean sprint times, together with a significant decrease in fatigue index, indicating greater sprint consistency and recovery capacity across repeated efforts. The reduction in fatigue index further implies improved buffering capacity, glycogen resynthesis, and lactate clearance [48,49]. Similarly, aerobic performance improved, with participants covering greater distances during YYIR1, reflecting enhanced intermittent endurance capacity. Collectively, these adaptations suggest improved neuromuscular readiness, metabolic efficiency, and cardiovascular function, consistent with evidence that adequate sleep supports reaction time, force production, oxygen utilization, and recovery processes [1,6,44,45,46]. Notably, ratings of perceived exertion remained similar for both tests, aligning with previous findings that athletes may not perceive reductions in physiological strain despite objective performance gains [1,7,50,51,52].

The divergent performance responses observed in the females are likely attributable to sleep-induced alterations in autonomic regulation that favour physiological recovery and endurance over maximal sprint performance, as sleep interventions have consistently shown to reduce physiological stress and prioritize restorative processes over power-oriented outputs [1,12,53,54,55,56,57]. Improvements in sleep duration and quality are associated with increased parasympathetic (vagal) activity and reductions in sympathetic dominance, reflecting a shift in autonomic balance that enhances cardiovascular stability, metabolic restoration, and fatigue tolerance during repeated or prolonged exercise [8,56,57]. In this context, improved endurance-related outcomes and fatigue resistance are consistent with evidence showing that sleep primarily augments recovery capacity and aerobic or intermittent exercise performance rather than maximal neuromuscular output [1,19,58]. In contrast, reductions in repeated-sprint performance may reflect a transient dampening of acute neuromuscular drive, as heightened parasympathetic dominance is accompanied by lower sympathetic activation, reduced catecholamine availability, and decreased central nervous system excitability, all of which are critical for rapid force production and peak sprint performance [8,56,57].

This interpretation is supported by evidence demonstrating a bidirectional relationship between sleep and the autonomic nervous system, whereby autonomic state influences sleep initiation and stage transitions, and sleep, in turn, reinforces autonomic down-regulation that may persist into waking hours (see Trinder et al. [56]). Notably, female athletes may exhibit heightened responsiveness to sleep-related behavioural interventions, potentially due to sex-specific differences in autonomic regulation and fatigue processing, which could partly explain the stronger endurance-related benefits observed in this group [9,59]. Collectively, these findings suggest that sleep-focused interventions may enhance recovery processes, endurance capacity, and resistance to fatigue, while maximal sprint output remains highly sensitive to acute neural activation and arousal state rather than chronic recovery optimization [9,33].

Pre-exercise cortisol responses further support the beneficial effects of SHIP. Anticipatory cortisol concentrations were consistently lower following the intervention, particularly prior to high-intensity exercise, reflecting a dampened hypothalamic–pituitary–adrenal (HPA) axis response. This attenuation suggests that improved sleep quality enhanced athletes’ psychophysiological resilience to pre-competition stress. Elevated cortisol is known to increase catabolic activity, accelerate protein breakdown, impair glycogen resynthesis, and heighten neuromuscular fatigue, all of which can hinder readiness and performance capacity [60,61,62]. Conversely, sufficient sleep supports more stable circadian regulation, reduces sympathetic activation, and promotes a more adaptive endocrine profile [27,63,64]. The pronounced reduction in anticipatory cortisol among females is particularly noteworthy and aligns with established gender differences in both stress reactivity and vulnerability to sleep disruption [28,43,65]. These results collectively indicate that even short-term improvements in sleep hygiene can meaningfully modulate endocrine stress responses, reducing physiological strain before exertion and potentially facilitating more efficient recovery.

Adherence patterns revealed moderate to high engagement with behavioural strategies such as consistent sleep routines and stimulus control, while environmental and nutritional components showed greater variability. This aligns with evidence that sleep hygiene comprises diverse behavioural and environmental factors with differing levels of uptake and perceived importance [30]. Such variability supports the feasibility of a customizable SHIP, where tailoring recommendations enhances adherence and autonomy in behaviour change [30,66]. Targeted education aimed at improving environmental and nutritional practices may further strengthen intervention effectiveness [2,9,12].

The present findings demonstrate that clusters of sleep hygiene behaviours, rather than isolated practices, are meaningfully associated with anaerobic performance and physiological responses. Five behaviourally coherent clusters explained a substantial proportion of variance (74.99%), supporting the use of a clustered, higher-order framework when examining sleep hygiene–performance relationships.

At the group level, cluster-based regression models were significant for RAST best and mean times and heart-rate responses, with moderate to largely explained variance (adjusted R^2^ = 0.38–0.50). Stimulant and metabolic regulation (Cluster 3) and bedroom light and thermal environment control (Cluster 4) consistently predicted sprint and physiological outcomes, whereas sleep continuity and behavioural regularity (Cluster 5) demonstrated inverse associations with performance and heart-rate responses. These findings highlight physiological arousal regulation and environmental optimization as key mechanisms linking sleep hygiene to high-intensity performance, consistent with evidence supporting environmental optimization for recovery [1,12]. The negative associations observed for Cluster 5 may reflect compensatory or restrictive behavioural patterns under training or academic load, whereby rigid routines or limited napping constrain short-term performance despite potential long-term sleep benefits. This nuanced effect of behavioural regulation aligns with previous observations of mixed performance responses under fatigue or recovery pressure [14]. Nutritional and stimulant-related behaviours showed consistent, rather than variable, associations when examined at a clustered level, supporting literature describing complex interactions between diet, sleep, and performance regulation [67].

Gender-specific analyses revealed notably stronger and more consistent associations in females, with all models demonstrating significance and high explained variance (adjusted R^2^ up to 0.79). In females, environmental and physiological regulation clusters predicted sprint performance, heart-rate responses, perceived exertion, and YYIR1 performance, whereas males demonstrated fewer and more isolated associations. These findings reinforce evidence of sex-related differences in sleep physiology and behavioural responsiveness [25] and underscore the importance of individualized and sex-specific sleep hygiene strategies in applied sport settings [44].

Overall, the present findings align closely with a growing body of literature demonstrating that improved sleep quality positively influences both anaerobic and aerobic performance while reducing fatigue and stress-related physiological responses such as cortisol [1,7,8,47,53]. Recent systematic reviews demonstrate that even modest extensions of nightly sleep, by approximately one hour, are associated with improvements in athletes’ physical performance, cognitive functioning, and recovery markers [9,33,68]. Similarly, sleep optimization interventions have been shown to improve sprint performance, reaction time, neuromuscular function, and post-exercise recovery, reinforcing the critical role of sleep as a performance determinant [7,12,47]. Our results contribute further by demonstrating these benefits in dual-career university athletes, a group at particular risk for compromised sleep due to combined academic and training demands [35]. The consistent improvements observed across both RAST and YYIR1 suggest that sleep hygiene exerts a broad regulatory effect on multiple physiological systems rather than task-specific adaptations [69]. This integrative pattern aligns with contemporary models of sleep-performance interactions, which propose that sleep influences multiple physiological pathways, including autonomic regulation, endocrine balance, cellular repair, and neurocognitive performance [8,47,68,69].

Several limitations should be considered. Although participants were requested to maintain consistent dietary, training, sleep, and daily routines, this could not be tightly controlled, which may influence sleep and performance outcomes. The relatively small sample size, particularly within gender-specific subgroups, may limit statistical power and generalizability, especially for regression analyses. The reliance on self-reported sleep measures without objective assessments (e.g., actigraphy) introduces potential bias, while the short intervention duration limits conclusions regarding long-term adaptations. Additionally, field-based performance tests may be influenced by environmental and motivational factors, and the clustering of sleep hygiene behaviours may oversimplify complex interactions between individual components. Future research should employ larger samples, incorporate objective sleep monitoring, and extend intervention durations to assess sustained effects. Further studies could explore individualized sleep hygiene strategies, as well as their impact on sport-specific performance and cognitive outcomes.

In conclusion, sleep hygiene represents a modifiable factor with meaningful implications for athletic performance and recovery. The SHIP improved sleep quality, enhanced anaerobic and aerobic performance, and reduced anticipatory cortisol responses, without increasing perceived exertion. These findings support the integration of structured sleep hygiene education into athletic training programmes as a practical and non-invasive strategy to optimize performance and recovery, particularly for dual-career athletes.

## Figures and Tables

**Table 1:** Sleep Hygiene Components.

SHIP Component	Recommendation
1	Avoid all electronic stimulants from 20:00
2	If unable due to academic loads, avoid it 30 min before going to bed
3	Changed any electronic stimulants screen to a “cool” light setting from 19:00
4	Wear the blue-light filtering glasses before going to bed
5	Minimize excess light from 21:00 to 21:30 by only having a bed lamp on
6	Change the bed lamp to a low-wattage globe
7	Manipulate the room temperature to 19–20 °C
8	Avoid consuming caffeine or any supplements from 17:00
9	Have the last large meal at least two to three hours before sleep
10	Consume a glass of lukewarm milk before going to bed
11	Consume a glass of chamomile tea before going to bed
12	Take a warm bath or shower before going to bed
13	Wear eye masks whilst sleeping
14	Wear earplugs whilst sleeping
15	Remove any timing instruments (i.e., clock) from the room
16	Avoid any sleep disruptions (i.e., bathroom timings) by limiting fluid intake
17	Sleep at least 8 h by keeping to a fixed routine
18	Take a short nap (<30 min), and avoid napping from 14:00

## Data Availability

The raw data supporting the conclusions of this article will be made available by the authors on request.

## Supplementary Materials

The following supporting information can be downloaded at: https://www.mdpi.com/article/10.3390/sports14050179/s1, Table S1: Rotated component matrix for sleep hygiene recommendations.

## Abbreviations

The following abbreviations are used in this manuscript:

SHIP	Sleep Hygiene Intervention Protocol
RAST	Repeated Anaerobic Sprint Test
YYIR-1	Yo-Yo Intermittent Recovery Test 1
PSQI	Pittsburgh Sleep Quality Index
REM	Rapid Eye Movement
RPE	Rate of Perceived Exertion
HRmax	Heart Rate Maximum
ESg	Hedges G Effect Size
PCA	Principal Component Analysis
KMO	Kaiser–Meyer–Olkin
HPA	Hypothalamic–Pituitary–Adrenal
