# Supplementary material for: A 14-Day Sleep Hygiene Intervention Improves Aerobic Performance and Reduces Anticipatory Cortisol in University Soccer Players
Source: Sports (Basel). 2026 Apr 29;14(5):179. doi: 10.3390/sports14050179 (PMC13210537; doi:10.3390/sports14050179)
Supplement: Supplementary file 1 [file sports-14-00179-s001.zip › sports-4251963-supplementary.pdf]

Table S1. Rotated component matrix for sleep hygiene recommendations

Sleep hygiene recommendation	Component 1	Component 2	Component 3	Component 4	Component 5
Cool light electronic device setting	0.886				
Avoid electronic devices 30 min before bed	0.840				
Chamomile tea before bed	0.796				
Eye masks	0.691		0.479		
Blue-light filtering glasses (Sleepspec)	0.660		0.487		
Warm milk before bed		0.865			
Remove clocks from the room		0.741			
Ear plugs		0.640			
Avoid electronic devices from 19:00		0.547			
Avoid caffeine after 17:00			0.850		
Last meal timing (≥2–3 h before sleep)			0.787		
Dim lights after 21:00				0.884	
Low-watt bedside globe				0.823	
Room temperature optimisation				0.673	
Avoid sleep disruptions					0.833
Fixed bedtime routine					0.710
Warm bath or shower before bed	0.458		0.443		0.528
Avoid late-afternoon naps					0.416

Component 1: Pre-sleep light exposure and device management

Component 2: Sensory and environmental sleep buffering

Component 3: Stimulant and metabolic regulation

Component 4: Bedroom light and thermal environment control

Component 5: Sleep continuity and behavioural regularity
